# Grief: The Epidemic Within an Epidemic

**DOI:** 10.1177/1049909120978796

**Published:** 2020-12-07

**Authors:** Sarah Elizabeth Petry, Dalton Hughes, Anthony Galanos

**Affiliations:** 1 3065 Duke University, Durham, NC, USA; 2 12277 Duke University School of Medicine, Durham, NC, USA

**Keywords:** grief, COVID-19, policy, bereavement, epidemic, complicated grief

## Abstract

COVID-19 has not only dramatically changed the way we live, it has also impacted how we die and how we grieve. With more and more Americans dying in ICU settings, away from family, and more funerals being held virtually, the pandemic has seriously curtailed normal expressions of grief and cultural mourning. Given the CDC guidelines for funerals and social distancing, simple human touch is no longer a mitigating force against prolonged grief. So, while one epidemic has a face and a name, we point to a second, more silent yet potentially equally devastating one, unacknowledged grief, and emphasize how policy can be a current therapeutic. We can wait for a vaccine, but we can also act now through thoughtful policymaking that acknowledges this second epidemic.

The United States is facing at least 2 public health crises, one unquestioned, and the other more obscure: coronavirus and unmitigated grief. One causes one thousand deaths a day, more than 260,000 deaths overall, and the other has more insidious consequences for both physical and mental health. Coronavirus provides multiple medical and immunologic challenges that are easy targets for doctors to address, while grief seems to go unacknowledged and is exacerbated by the chronic mortality suffered daily in our country. The second health crisis, grief, may be inadequately addressed, but it is one shared certainty in these uncertain times. If we take a step back to acknowledge this cumulative burden of grief as a result of COVID-19, what can we do to mitigate or soften its impact?

COVID-19 has caused immeasurable loss in so many areas of life. Loss is deeply tied to rituals of grief, not only in terms of grieving the death of loved ones, but also grieving the loss of work or opportunities or normalcy. As more people die, survivors must grieve without traditional rituals, such as memorial services, that could enable healthy coping and prevent negative health outcomes in the long run. To prevent poor health outcomes for the thousands of bereaved Americans, we must first acknowledge this growing epidemic of grief. In this paper we explore the extent to which people are able to express their grief and the ways in which policies might exacerbate or alleviate consequences of grief both in this moment and beyond it.

## Grief & Bereavement

Bereavement is traditionally defined as losing a loved one to death, while grief encompasses emotional reactions to bereavement.^1,2^ Some losses are a normal part of the life course, such as death of one’s parents during adulthood.^1^ Even “normal” losses, though, and the accompanying grief, can have deleterious health consequences. For example, bereavement is associated with an increased risk of mortality and greater likelihood of poor physical health, particularly among recently bereaved individuals.^1,2^ In addition, the psychological effects of bereavement are diverse and vary in intensity based on the type of lost relationship and the timing of loss.^2^

Complicated grief is a prolonged grief experience, lasting 6 months or more, often following losses that are not considered a typical life course event.^1,2^ This could be due to the timing of loss in one’s life, the intensity of the relationship that was lost, or underlying psychological conditions.^1^ Black Americans are more likely to experience loss early in life and more cumulative losses across the life course.^3^ This means that not only are Black Americans more likely to experience loss, but, though data are limited, Black Americans would seem to be at higher risk for complicated grief. Both complicated and uncomplicated grief are associated with multiple adverse psychological reactions, some of which are short-term, others of which are experienced over many years.^1^ Physicians, even palliative care physicians, who often work with dying patients, lack adequate education on grief and bereavement, limiting their ability to respond to these universal experiences.^4^

Unlike complicated grief, which captures an emotional reaction to loss,^1^ disenfranchised grief captures the psychological, sociological, and political aspects of loss.^5^ In particular, disenfranchised grief describes the experience of grieving losses that are unacknowledged or unsupported by social systems.^5^ This definition offers a role for the state in mitigating the potentially deleterious effects of complicated grief. In the US, as people continue to lose loved ones to COVID-19, disenfranchised grief may be more prevalent: there are few socially supported mechanisms for grieving, and little state support for bereaved citizens,^6^ compounding the negative psychological consequences of grief.

Given the immense death toll of the coronavirus, many people in the US (and around the world) are experiencing what has become an acute convergence of loss. Social support can ameliorate negative health outcomes that accompany bereavement and grief.^1^ Moreover, there are common practices following bereavement that involve increased contact with family and friends, ritualistic gatherings, and general support from existing social relationships.^7^ Although mourning rituals vary across cultures and places, these rituals can provide bereaved individuals with feelings of control and relief in times that feel chaotic.^7^ For many, reliance on social supports and engaging in rituals of mourning are sufficient to help those who are grieving avoid complicated grief and return to some normalcy in their own lives.^7^ However, current

Unprecedented conditions—massive numbers of casualties; forced separations during a patient’s final days; and denial of physical touch, final goodbyes, and traditional mourning rituals—pose threats to bereaved family members’ mental health, leaving them vulnerable to intense and enduring psychological distress.^8^

Today, people are encouraged to socially distance themselves from others who do not reside in their own homes. Although we are increasingly connected to one another through technology, our traditional forms of social support are limited given these restrictions, suggesting that our ability to rely on them to ride out the waves of grief are limited, too. Not only that, but mourning rituals have changed in the face of COVID-19. The CDC recommends limiting the size of funeral services, the largest rituals, or using video conference technology in lieu of physical attendance at such gatherings.^9^ Engaging in ritual can, by itself, mitigate grief, but it is unclear whether virtual connection and virtual rituals serve the same healing and protective purpose as in-person social support and ritualized behavior.^7^

Americans are trying hard, however, to continue important rituals. During the pandemic, an author on this manuscript has held a virtual funeral service to commemorate the loss of a parent. Using the virtual format allows for greater attendance in a memorial service as there is no need to travel to an individual location. This translated to over 100 participants in the conference dispersed over the United States, Canada, as well as several countries in Europe and Africa. Since the use of video conferencing is widespread, members were able to log on via computers, cell phones, and tablets and play an active role by sharing stories with each other. Additionally, the associated costs surrounding a funeral service are heavily decreased as it is no longer necessary to rent a central location or accommodate supplemental amenities. And yet despite these perceived advantages, the lack of the traditional funeral was felt. Personal forms of support that we know mitigate grief—the in-person wishes and the gestures of respect, not to mention the old fashioned “hug”—were simply not there.

The more than 260,000 people who have died of COVID-19 in the US have left thousands struggling to grieve and mourn within the new confines of a pandemic world.^8^ According to one of those survivors,

Our rituals of grief are no more. These are now mediated through distance and must emerge in new forms as we feel the cut-me-to-the-core pain of grief in isolation…We yearn for human comfort, yet we know all too well the excruciating cost that lifting distance too early could engender.^10^

Human contact provides an essential component of mourning rituals. Yet it is precisely that component that is out of reach due to this pandemic.

Finally, COVID-19 has led to bereavement not only in response to loss of family members and friends, but also loss of jobs, social proximity, and so much more. For those who are grieving a loss of any kind, it is likely that their experience will be magnified by both the restrictions on social gatherings in place for the sake of public health, and the intersection of their grief over many losses. This is acutely true within communities of color, who face a greater burden of loss from COVID-19 as well as a higher cumulative burden of bereavement across the life course.^3^ Collectively, the US and the world are grieving the loss of connectivity, normalcy, and hundreds of thousands of lives. It is unclear to what extent the necessary restrictions will preclude healthy coping among survivors. But both within and outside this unprecedented time, there are ways that policies might alleviate the strains of loss, bereavement, and grief.

## Policies to Address a Grieving World

Policies, such as paid time off and economic support payments, can promote healthy grieving and can prevent complicated or disenfranchised grief.^5,7^ Disenfranchised grief is distinct from complicated grief insofar as it explicitly refers to grief experienced without acknowledgment, public mourning or social support.^5^ Lack of social support, and disenfranchisement, more generally, can then foster complicated grief reactions.^2^ Unlike complicated grief, though, disenfranchised grief situates a psychological phenomenon within a social and political context, identifying the role that policy and government might play in facilitating or precluding healthy coping behaviors.^5^ An institution, such as the state or a company, can provide paid time off or economic benefits to bereaved individuals as an intentional recognition of their role in mitigating grief.

In the US, though, even federal policies designed to enable individuals to take time off to care for a loved one, such as the Family Medical Leave Act, do not allow leave to be taken when bereavement—the death of a loved one—ultimately occurs.^1,6^ This lack of institutional support for bereaved individuals in the workplace devalues the experience of grief and precludes healthy mental and physical responses.^5^ Employers may establish their own policies, though these typically allow only 3 to 4 days of paid bereavement leave.^6^ The US falls behind other developed countries, where, unlike the US, they implement policies that attempt to assuage disenfranchised and complicated grief by acknowledging and supporting bereaved citizens.

Other countries have policies to address the varied needs of bereaved citizens. In the UK, for example, the Bereavement Support Payment program provides bereaved spouses lump sum payments over 18 months to address the financial consequences of losing a spouse or civil partner.^5^ However, much like the US, the UK does not have mandated bereavement leave policy, though employers might establish their own.^5^ In Israel, there are 3 lump sum payment programs for bereaved citizens, funeral services are essentially paid for by the government, and all employees are entitled to 3 to 7 days paid bereavement leave.^5^ These policies, though imperfect, demonstrate that these countries recognize bereavement as a social and political phenomenon,^5^ and recognize the role that government can play in addressing the needs of bereaved citizens.

Colleges and universities might also offer insight into how to leverage policies to provide bereavement support, though bereavement leave policies are relatively uncommon.^11^ In evaluating the Grief Absence Policy for Students (GAPS), students at one large Midwestern university reported that the presence of this program was an important aspect of a broader system of support.^11^ Simply knowing they could take time off if needed and seek additional resources, such as counseling, during periods of bereavement offered students peace of mind.^11^ Students reported that taking that allotted time off also provided psychological relief.^11^

Although examples of bereavement policy are limited, adapting and implementing some interventions is critical to address the epidemic of grief within the COVID-19 epidemic. The pandemic has been ongoing for months, yet policy makers still have not entirely acknowledged the diverse needs of the many people experiencing grief and bereavement. Rituals of mourning and social support have both been curtailed, if not eliminated, and even where virtual options exist, as a coauthor on this paper notes, they lack many of the tangible comforts associated with in-person gatherings. Disenfranchised grief thrives in exactly these circumstances,^5^ and complicated grief is likely to follow.^2^

Grief is social and it has now become embedded in a political context. Ignoring bereavement, which more Americans experience every day, and which is compounded by numerous non-human losses and ceaseless uncertainty, inhibits grief, coping, and healing. Bereavement leave, increased welfare supports, such as those in place in the UK and Israel, and better resources for bereaved individuals and families might mitigate these crises to a point. Without a treatment or vaccine, perhaps one avenue of therapeutic benefit in the present is for policy makers, clergy, and citizens to acknowledge this grief and support its expression. This might involve offering advice about the ways that social support prevents many negative health outcomes associated with grief. Some hospital systems have gone a step further and implemented bereavement hotlines, connecting bereaved individuals to palliative care and behavioral health specialists,^12^ while many more have enabled virtual connections between their dying patients and loved ones at home.^10,12^ These programs offer a blueprint for how to provide critical resources, even when limited by necessary public health guidelines.

Addressing the needs of bereaved Americans is essential to preventing further public health crises, when those without the resources and support they need are more likely to experience complicated grief and the many negative psychological and physical health outcomes associated with that experience.^1^ Failing to acknowledge this epidemic of grief will, by definition, result in disenfranchised grief.^7^ Instead, acknowledgment “is the only real medicine of grief.”^13^ Therefore, policy makers must first recognize this growing group experiencing complicated grief as a direct result of the many losses associated with COVID-19. Bereavement policy must be flexible, providing a toolkit of resources that can address the diverse downstream physical and mental health consequences of grief.^1,2^ Policy must acknowledge that bereavement will look different today, because, for all of us, it is complicated by so many other losses and experiences of grief.

## Summary: Policy as Another Therapeutic

While there are many facets to this pandemic, we raise the issue of grief and its public health implications as our death toll rises and rises. And if, by definition, this virus and subsequent limitations on social contacts and diminshed grief rituals, complicates our grief and exacerbates the negative outcomes, then we need to acknowledge the epidemic within the epidemic. Waiting for a vaccine has shown that the graphs of mortality just keep climbing. We urge policymakers to acknowledge the pervasive grief and its many consequences. Beyond recognition, policymakers can take tangible steps *now* to stem the flow of grief following so much loss.

For now, policy must address the needs of those who are unable to access support and rituals due to current restrictions. One way to do this would be through primary preventive interventions, which would make bereavement counseling available to anyone who wants it.^1^ Interventions of this sort have been proven to be effective at reducing the negative health outcomes of bereavement when the bereaved person is able to decide for themselves if they want to pursue counseling.^1^ This will require funding and online infrastructure, as well as coordinated efforts to disseminate the availability and benefits of such a program.

Perhaps we can follow the lead other countries, such as the UK or Israel, who recognize that grief and bereavement are embedded in social and political contexts.^5^ Perhaps just knowing that we acknowledge the grief, and that we are being intentional, will offer some basic support as the GAPS program did for college students.^11^ Until a stronger solution like a vaccine arrives, then it is policy, both now and later that can help immunize and soften the grief that is a hidden epidemic within an epidemic.
